# Peroxynitrite detoxification by *Trypanosoma cruzi* heme peroxidase supports parasite survival in macrophages

**DOI:** 10.1016/j.jbc.2025.110533

**Published:** 2025-07-28

**Authors:** Vera Skafar, Matilde Abboud, Samuel L. Freeman, Alejandra Martínez, Emma L. Raven, Rafael Radi, Lucía Piacenza

**Affiliations:** 1 Departamento de Bioquímica, Facultad de Medicina, Universidad de la República, Montevideo, Uruguay; 2 Centro de Investigaciones Biomédicas (CEINBIO), Facultad de Medicina, Universidad de la República, Montevideo, Uruguay; 3 School of Chemistry, University of Bristol, Bristol, UK

**Keywords:** Chagas disease, *Trypanosoma cruzi*, heme peroxidase, macrophages, oxidants, peroxynitrite, ascorbate

## Abstract

*Trypanosoma cruzi*, the causative agent of Chagas disease, must overcome a host’s nitro-oxidative burst to establish the infection. Among the reactive species generated by immune cells, peroxynitrite stands out as a highly cytotoxic molecule for the parasite. Yet, the parasite’s defense mechanisms against peroxynitrite remain incompletely understood. In this work, we demonstrate that the *T*. *cruzi* heme-containing ascorbate peroxidase cytochrome c peroxidase (APx-CcP), an antioxidant enzyme previously shown to increase parasite infectivity, acts as a key peroxynitrite-detoxifying enzyme. Using direct and competitive kinetic assays, we show that APx-CcP reacts rapidly with peroxynitrite (*k* = 3–4 × 10^6^ M^-1^ s^-1^ at pH 7.4 and 25 °C) *via* a two-electron reduction mechanism. This activity allows *T*. *cruzi* to neutralize peroxynitrite within the macrophage phagosome, increasing survival and infection capacity, particularly in strains with elevated APx-CcP expression. This is the first heme peroxidase of trypanosomatids in which a catalytic cycle of peroxynitrite decomposition is described. Moreover, the presence of physiological concentrations of ascorbate during infection enhances the enzyme's ability to scavenge both hydrogen peroxide and peroxynitrite, further highlighting its role in parasite survival.

Chagas disease, caused by the protozoan parasite *Trypanosoma cruzi* (*T*. *cruzi*), affects 6 to 7 million people worldwide and remains a major public health challenge (https://www.who.int/news-room/fact-sheets/detail/chagas-disease-(american-trypanosomiasis). While the acute phase is often asymptomatic or mild, up to 30% of infected individuals progress to chronic forms with severe cardiac or digestive complications (1). Current therapies—benznidazol and nifurtimox—are limited by their toxicity, variable efficacy, and inability to fully prevent chronic disease progression, highlighting an urgent need for novel therapeutic targets (2, 3).

Macrophages represent the first line of defense in early infection, recognizing and engulfing *T*. *cruzi via* phagocytosis (4), with the activation of NADPH oxidase (NOX-2) and the production of high levels of superoxide radical anion (O_2_^•-^) directed to the internalized parasite (4, 5, 6). Additionally, immune-stimulated macrophages (expression of the nitric oxide synthase (iNOS)) produce nitric oxide (^•^NO) that combines fast with O_2_^•-^ (k ∼ 10^10^ M^-1^ s^-1^ (7)), leading to peroxynitrite (the term peroxynitrite is used to refer the sum of peroxynitrite anion and acid peroxynitrous, ONOO^-^ and ONOOH, respectively), a more potent cytotoxic agent against the parasite (5, 6). *T*. *cruzi* relies on a unique and robust antioxidant system that differs from that of its mammalian host, rendering attractive targets for pharmacological intervention (8, 9, 10, 11, 12, 13).

One of the antioxidant enzymes is ascorbate cytochrome c peroxidase (APx-CcP), a heme-containing enzyme that scavenges H_2_O_2_ using ascorbate or cytochrome c as a reducing substrate (14, 15, 16, 17, 18). TcAPx-CcP is localized not only to the endoplasmic reticulum and mitochondria, but also to the plasma membrane in infective stages (14). Given the high concentrations of ascorbate, both in the host and parasite (19, 20, 21), this dual-substrate versatility may represent an evolutionary adaptation (22). Peroxynitrite reacts with hemeproteins through diverse mechanisms. Some react *via* a two-electron mechanism, leading to the formation of compound I and NO_2_^-^ (such as horseradish peroxidase (HRP), catalase, and prostaglandin endoperoxide H synthase-1) (23, 24, 25). Others react through a one-electron mechanism, forming Compound II and ^•^NO_2_ (such as myeloperoxidase (MPO), lactoperoxidase (LPO), and chloroperoxidase (CPO)) (26, 27, 28). Finally, some hemeproteins catalyze the isomerization of peroxynitrite to NO_3_^-^ (as is the case with oxy-, methemoglobin, and metmyoglobin) (29). The reactivity of TcAPx-CcP with peroxynitrite remains unexplored and may reveal novel insights into its protective role during infection. In this study, we investigated the role of TcAPx-CcP in peroxynitrite detoxification, uncovering mechanistic features that underline its importance in the antioxidant defense of *T*. *cruzi* and establishing the relevance of its activity for parasite survival during macrophage infection.

## Results

### APx-CcP reacts fast with peroxynitrite

**Figure 1:**
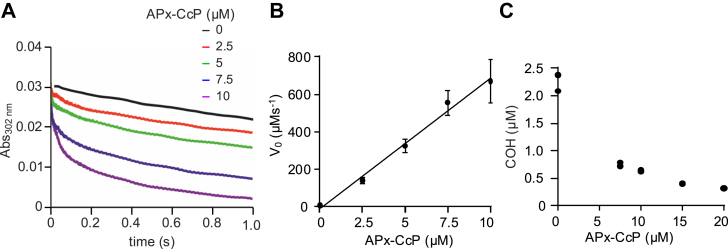
**Recombinant APx-CcP reacts fast with peroxynitrite**. *A*, time courses following absorbance at 302 nm corresponding to peroxynitrite decomposition (15 μM) in the presence of increasing concentrations of APx-CcP (0–10 μM). A linear function was fitted to the first 10 ms of the reaction and the initial rate values calculated from the slopes. *B*, initial rate values *versus* APx-CcP concentration. A second order rate constant of 4 x 10^6^ M^-1^s^-1^ at pH 7.4 and 25 °C was calculated from the slope. *C*, competitive kinetic assay with coumarin boronic acid (CBA). The concentration of coumarin (COH, quantified by HPLC) the product of the reaction of peroxynitrite with CBA is plotted against APx-CcP concentration (0–20 μM). A second order rate constant of 3 x 10^6^ M^-1^s^-1^ was calculated at pH 7.4 and 25 °C.

The reactivity of APx-CcP with peroxynitrite was evaluated by two different methods. First, using a direct method (stopped flow), a second-order rate constant of 4 x 10^6^ M^-1^ s^-1^ for the reaction was determined at pH 7.4 and 25°C (Fig. 1, *A* and *B*). Alternatively, the reaction was studied by a competition kinetic assay using coumarin boronic acid (CBA). This compound reacts directly with peroxynitrite (*k* = 1.1 x 10^6^ M^-1^ s^-1^ at pH 7.4 and 25 °C), forming a fluorescent product 7-hydroxycoumarin (COH) that can be measured by HPLC (30). As expected, increasing concentrations of APx-CcP inhibited peroxynitrite-mediated COH formation (Fig. 1*C*). The second-order rate constant calculated by this method was 3 x 10^6^ M^-1^ s^-1^ at pH 7.4 and 25 °C, which is in excellent agreement with the value estimated by the direct method. Notably, the rapid reaction of APx-CcP with peroxynitrite is comparable to the fastest heme peroxidases, with rate constants in the range of 10^6^-10^7^ M^-1^s^-1^ (31).

### Detection of enzyme intermediates in the reaction with peroxynitrite

**Figure 2:**
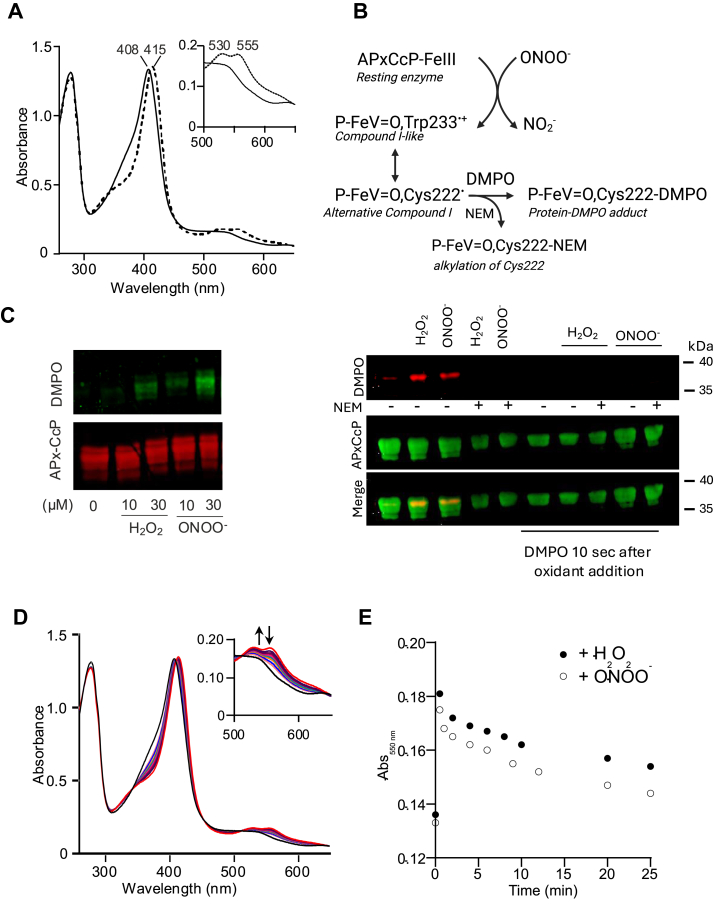
**Detection of enzyme intermediates and peroxynitrite-mediated inactivation of recombinant APx-CcP**. *A*, UV-visible spectra of APx-CcP before (*solid line*) and after peroxynitrite addition (*dashed line*). Oxidant was added in an oxidant:enzyme ratio of 1.5:1. Spectral changes indicate the formation of a compound I-like (FeV=O,Trp233^•+^) intermediate. *B*, schematic representation of the proposed mechanism of reaction. APx-CcP reacts with peroxynitrite *via* a two-electron mechanism, yielding a compound I-like species and NO_2_^-^. An “alternative” compound I intermediate (FeV=O,Cys222^•^) is trapped in the presence of DMPO (protein-DMPO adduct). Adduct formation is prevented when the enzyme is pre-treated with NEM (FeV=O,Cys222-NEM). The protein-DMPO adduct can be detected by Western blot with an anti-DMPO antibody. *C*, *Left panel*, APx-CcP (10 μM) was exposed to H_2_O_2_ or peroxynitrite (0, 10 or 30 μM) in the presence of DMPO (100 mM), followed by Western blot analysis with anti-DMPO (*green*) and anti-APx-CcP (*red*) antibodies. *Right panel*, APx-CcP (10 μM) was exposed to H_2_O_2_ or peroxynitrite (30 μM) in the presence or absence of NEM (50 mM). As indicated in the figure, DMPO (100 mM) was present before or added 10 s after peroxynitrite addition, Western blot anti-DMPO and anti-APxCcP are shown in *red* and *green* respectively. *D*, UV-visible spectra of APx-CcP exposed to peroxynitrite (1:1 ratio) recorded at different time points after oxidant addition (0–25 min). *E*, Absorbance at 550 nm over time of APx-CcP exposed to H_2_O_2_ or peroxynitrite (ONOO^-^) in a 1:1 ratio.

When working with heme peroxidases and their reaction with peroxynitrite, it is not possible to generalize about the enzyme intermediates that are formed in the catalytic cycle (32). They must be studied case by case and traditionally, they have been distinguished based on their spectroscopic properties. In APx-CcP, three enzyme intermediates have been identified during the catalytic cycle: Compound I (FeIV=O,P^•+^), Compound I-like (FeIV=O,Trp233^•+^), and Compound II (FeIV=O) (14, 22). To detect these intermediates, recombinant APx-CcP was exposed to peroxynitrite, and UV-vis spectra were recorded from 250 to 650 nm (Fig. 2*A*). The spectrum of the resting enzyme had the Soret peak at 408 nm that shifted to 415 nm after the reaction with peroxynitrite, with the appearance of two bands at 530 and 555 nm. These spectral changes resemble those observed in the reaction of TcAPx-CcP with H_2_O_2_ (14) corresponding to the two-equivalent oxidized enzyme intermediate compound I-like (FeIV=O,Trp233^•+^).

To gain further insight into the nature of the enzyme intermediate being formed, we performed immuno-spin trapping experiments using the spin trap 5,5-dimethyl-1-pyrroline-N-oxide (DMPO) that forms stable protein-nitrone adducts after its reaction with protein radicals. These adducts can be detected with specific anti-DMPO-nitrone antibodies (33). In previous studies, this technique supported the formation of a transient “alternative” compound I with a thiyl radical in residue Cys222 of TcAPx-CcP after reaction with H_2_O_2_ (FeIV=O, Cys222^•^, see scheme in Fig. 2*B*) (14, 22). Theoretical calculations of the electron transfer pathways in TcAPx-CcP indicated that both pathways (Trp233-heme and Cys222-heme) would be probable (34). Either Cys alkylation and/or the C222A mutant lost their ability to use cytochrome c as the reducing substrate, suggesting that Cys222^•^ formed after oxidant reaction is important for the stabilization/protection of Trp233^•+^, a key residue for enzyme activity (14, 22).

Consistent with these findings, when APx-CcP was exposed to peroxynitrite in the presence of DMPO, the formation of an “alternative” compound I was detected in a dose-dependent manner (Fig. 2*C*, left panel). As expected, the trapping of the cysteinyl radical by DMPO was inhibited in the presence of N-ethylmaleimide (NEM), an alkylating agent that reacts with sulfhydryls to form stable thioethers (Fig. 2*C*, right panel). Interestingly, when DMPO was added 10 s after oxidant addition, no signal was detected, confirming its transient nature (22). In contrast, the compound I-like species (FeIV=O, Trp233^•+^) formed after reaction with either H_2_O_2_ or peroxynitrite remains relatively stable over time. The endogenous decay was monitored by absorbance decrease at 550 nm, presenting an estimated half-life of 6 min (Fig. 2, *D* and *E*).

For HRP and prostaglandin endoperoxide H synthase, peroxynitrite reacts *via* a two-electron mechanism, yielding compound I and nitrite (NO_2_^-^) as final products. Given that a compound I-like intermediate was detected in APx-CcP after reaction with peroxynitrite, we measured the production of NO_2_^-^ using the Griess method to confirm a similar mechanism. The yield of NO_2_^-^ was approximately in a 1:1 ratio, supporting a two-electron oxidation of APx-CcP (Fig. 2*B* and Table S1).

Finally, we assessed whether APx-CcP is susceptible to inactivation and tyrosine nitration in the presence of peroxynitrite. Notably, the enzyme proved to be highly resistant to oxidant-dependent inactivation and nitration, retaining 85% of its activity after exposure to a 10-fold molar excess of peroxynitrite (Fig. S2).

### APx-CcP catalytically detoxifies peroxynitrite using ascorbate as a reducing substrate

**Figure 3:**
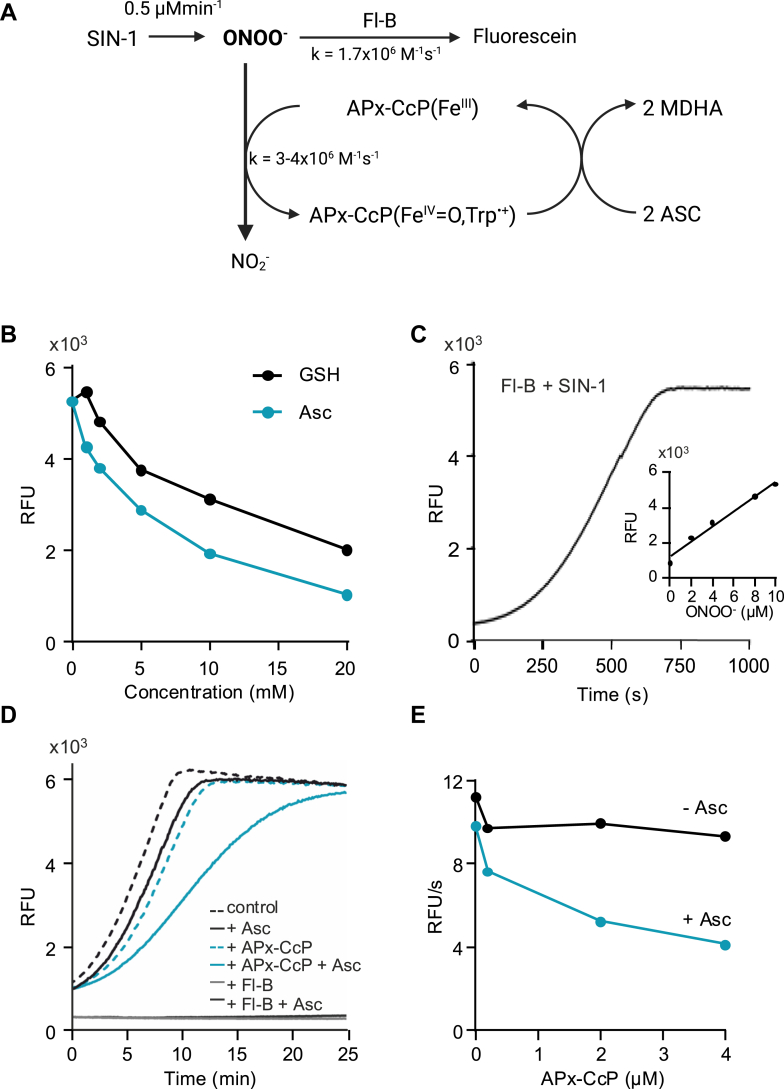
**APx-CcP catalytically detoxifies peroxynitrite using ascorbate as a reducing substrate**. *A*, schematic representation of the competition assay used in *panel d*, (MDHA: monodehydroascorbate radical). *B*, Fl-B (10 μM) was exposed to peroxynitrite (5 μM) in the presence or absence of ascorbate and/or GSH (0–20 mM) and fluorescein formation was followed fluorometrically (λexc = 492 nm, λem = 515 nm). *C*, a flux of peroxynitrite was generated using SIN-1 (0.5 mM, 50 mM phosphate buffer, pH 7.4) and the peroxynitrite flux was estimated using a calibration curve with Fl-B and known peroxynitrite concentrations (∼0.5 μM/min, 25 °C, inset). *D*, time courses for the oxidation of Fl-B by SIN-1 in the absence (*black dashed line*) or presence of ascorbate (Asc, 200 μM, *solid black line*). Oxidation of Fl-B was also assessed in the presence of APx-CcP (2 μM) in the absence (*blue dashed line*) or presence of ascorbate (*blue solid line*). *E*, the slopes (*linear region*) obtained in *panel d* for different APx-CcP concentrations (0–4 μM) were plotted against enzyme concentration in the absence or presence of ascorbate (Asc). RFU, Relative fluorescence unit.

The catalytic activity of APx-CcP with ascorbate was evaluated using a competition assay in the presence of ascorbate, peroxynitrite, and fluorescein boronate (Fl-B) (35). FlB reacts directly with peroxynitrite to form the fluorescent product fluorescein (1.1 x 10^6^ M^-1^ s^-1^ at pH 7.4 and 25 °C). The experimental design is summarized in Figure 3*A*, and conditions optimized considering the known reactivity of peroxynitrite with ascorbate (∼10^2^ M^-1^ s^-1^) (36, 37, 38). A concentration of 200 μM ascorbate was selected, as higher concentrations (>1 mM) were found to directly inhibit peroxynitrite-mediated Fl-B oxidation (Fig. 3*B*). A flux of peroxynitrite (∼0.5 μM/min) was generated using SIN-1 (Fig. 3*C*), and peroxynitrite-dependent Fl-B oxidation was followed fluorometrically over time. As shown in Figure 3, *D* and *E*, increasing concentrations of APx-CcP inhibited fluorescein formation, with the inhibition being markedly more pronounced in the presence of ascorbate (Fig. 3, *D* and *E*). These results unequivocally demonstrate that APx-CcP can catalytically decompose peroxynitrite in the presence of one of its reducing substrates.

### Detoxification of macrophage-derived peroxynitrite

**Figure 4:**
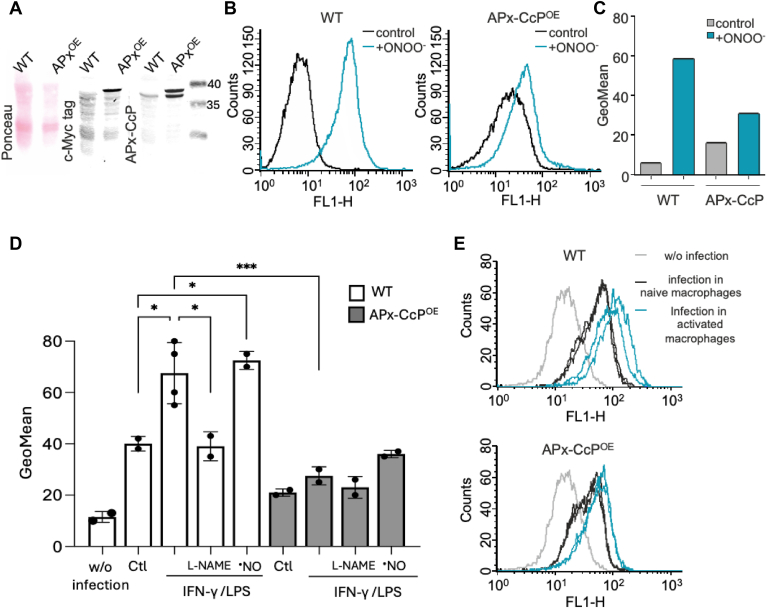
**Peroxynitrite detoxification by APx-CcP overexpressing parasites**. *A*, ponceau staining and Western blot analysis of WT and APx-CcP overexpressing parasites (APxCcP^OE^) extracts (1 x 10^7^ cells) with anti-cMyc9E10 and anti-APx-CcP antibodies. *B*, Fl-B pre-loaded epimastigotes (WT, *left* and APx-CcP^OE^, *right*) were exposed to a single bolus addition of peroxynitrite (25 μM) and intracellular Fl-B oxidation was evaluated by flow cytometry. *C*, geomean of Fl-B oxidation in each condition tested. *D*, culture-derived trypomastigotes from WT- and APx-CcP^OE^ parasites were pre-loaded with Fl-B and used to infect naïve or immunostimulated macrophages at a parasite:cell ratio of 10:1. Geomean representation of intraphagosomal Fl-B oxidation under the indicated experimental conditions. Ctl, naive macrophages; L-NAME (10 mM) was added to macrophage cultures 30 min before infection; NOC-18 (1 mM, ^•^NO) was used as a positive control. Data are means ± S.D of at least three independent determinations. *E*, representative flow cytometry analysis of Fl-B oxidation in WT (*upper* figure) or APx-CcP^OE^ (*lower* figure) parasites in non-infected (w/o infection); infected naïve and infected immunostimulated macrophages. ∗, ∗∗ Denotes statistically differences (*p* < 0.05; *p* < 0.005) by one-way ANOVA and post Tukey´s multiple comparison test.

Once we confirmed that the recombinant enzyme catalytically detoxifies peroxynitrite in a cell-free system, we assessed the relevance of this reaction in parasites using Fl-B, previously validated for the detection of endogenously produced peroxynitrite (10, 35). First, parasites overexpressing APx-CcP (APx-CcP^OE^ parasites), which exhibited ∼7-fold increase in APx-CcP content compared to wild type (CL-Brener, WT) (Fig. 4*A*). Then, WT (control) and APx-CcP^OE^ epimastigotes were pre-loaded with Fl-B, and a single dose of peroxynitrite (25 μM) was added to the parasite suspensions, followed by evaluation of intraparasite Fl-B oxidation by flow cytometry (Fig. 4*B*). As shown in Figure 4C, WT parasites exhibited higher Fl-B oxidation compared to APx-CcP^OE^ parasites.

We next aimed to evaluate Fl-B oxidation in the infective trypomastigote stage of the parasite during macrophage infections. For this, naive- or interferon-gamma/lipopolysaccharide- (IFN-γ-LPS) stimulated macrophages (iNOS induction and thus peroxynitrite generation) were infected with Fl-B pre-loaded trypomastigotes at a parasite: macrophage ratio of 10:1, and Fl-B oxidation within the phagosome was measured by flow cytometry 1 hour post-infection (Fig. 4, *D* and *E*).

In WT parasites, Fl-B oxidation was detected upon macrophage phagocytosis, likely due to NOX-2 activation and the subsequent production of O_2_^•^^-^/H_2_O_2_. Infections performed in immunostimulated macrophages exhibited even higher Fl-B oxidation, which was significantly inhibited by L-NAME (an iNOS inhibitor), confirming the intraphagosomal generation of peroxynitrite (Fig. 4, *D* and *E*, blue histograms). Additionally, a nitric oxide donor (NOC-18, ^•^NO) was used as a positive control for peroxynitrite generation, yielding comparable results.

Notably, in all experimental conditions tested, Fl-B oxidation was markedly lower in APx-CcP^OE^ than in WT parasites. These results demonstrate that APx-CcP effectively detoxifies macrophage-derived H_2_O_2_ and peroxynitrite *in cellula*, protecting *T*. *cruzi* from oxidative stress within the host environment.

### Extracellular ascorbate supports parasite peroxynitrite detoxification during macrophage infection

**Figure 5:**
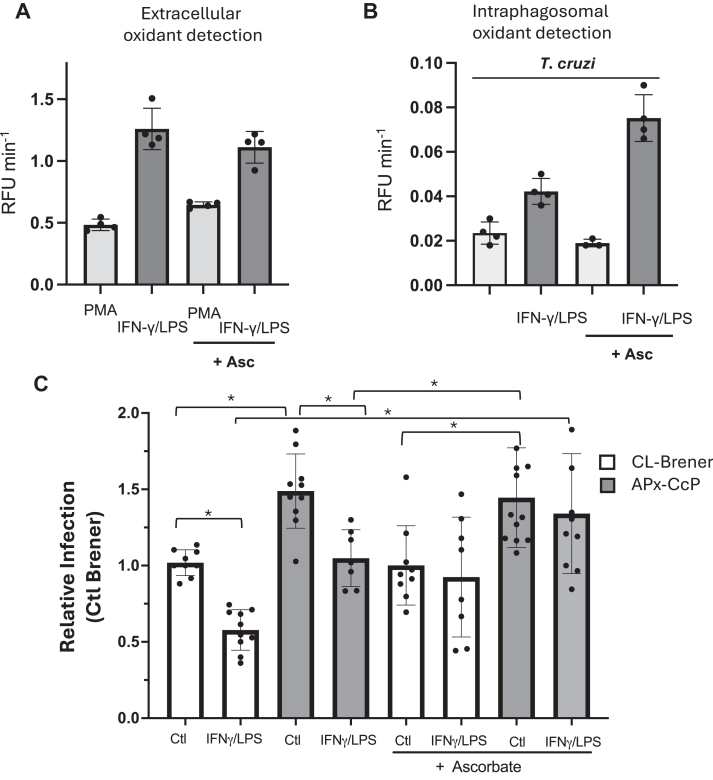
***T*. *cruzi* macrophage infection in the presence of ascorbate**. *A*, macrophages (naïve or immunostimulated for 5 h) were incubated in dPBS containing Fl-B (50 μM) in the presence or absence of ascorbate (200 μM). Extracellular O_2_^•-^ production was elicited with PMA (4 μg/ml) and Fl-B oxidation followed fluorometrically at λexc = 492 nm and λem = 515 nm (Varioskan). Results are expressed as relative fluorescence units per second (RFU/s). *B*, Fl-B pre-loaded culture derived trypomastigotes (parasite:cell ratio 10:1) were used to infect naïve or immunostimulated macrophages in the presence or absence of ascorbate (200 μM) and Fl-B oxidation followed as in *panel A*. *C*, *T*. *cruzi* macrophage infection at 24h in naïve and/or immunostimulated macrophages in the presence or absence of ascorbate (200 μM). Results are the means of at least four independent experiments each per duplicate and are expressed relative to WT in the control condition (naïve macrophages). Data are means ± SD of at least three independent determinations. ∗ Denotes statistically differences (*p* < 0.05) by one-way ANOVA and post Tukey´s multiple comparison test.

To further investigate the role of extracellular ascorbate in *T*. *cruzi* resistance to macrophage-derived oxidants, we performed infection assays using CL-Brener (WT) and APx-CcP^OE^ trypomastigotes in naive and/or immunostimulated macrophages in the presence or absence of extracellular ascorbate. We first tested whether ascorbate could directly scavenge H_2_O_2_ and ONOO^-^ in the extracellular medium. Using the fluorescent probe Fl-B, macrophages were stimulated with phorbol 12-myristate 13-acetate (PMA) to elicit O_2_^•^^-^/H_2_O_2_ or PMA + IFN-γ/LPS (to induce peroxynitrite generation), and Fl-B oxidation was monitored. Ascorbate (200 μM) did not inhibit Fl-B oxidation under either condition, indicating that the vitamin did not scavenge these oxidants in the culture media (Fig. 5*A*). Similarly, when Fl-B-preloaded trypomastigotes were used to detect intraphagosomal oxidants, extracellular ascorbate at the tested concentrations did not affect Fl-B oxidation, ruling out direct scavenging effects in this experimental setup (Fig. 5*B*). Intriguingly, intraphagosomal Fl-B oxidation in immunostimulated macrophages was higher in the presence of extracellular ascorbate. This is likely due to increased ^•^NO production by membrane-bound iNOS (39) driven by the ascorbate-dependent elevation of tetrahydrobiopterin levels, a key cofactor for NOS activity (40).

We then evaluated whether *T*. *cruzi* could use extracellular ascorbate as a reducing substrate for APx-CcP-mediated peroxynitrite detoxification. Ascorbate was added at the onset of infection, and parasite survival was evaluated 24 h post-infection. In naïve macrophages, APx-CcP^OE^ parasite showed higher survival than WT, consistent with their enhanced capacity to detoxify NOX-2-derived H_2_O_2_ (14). However, ascorbate addition had no further effect on survival in this setting (Fig. 5*C*), likely because of the non-limiting endogenous ascorbate pool in the parasite cytosol and the presence of additional H_2_O_2_-scavenging enzymes (peroxiredoxins). Under conditions promoting peroxynitrite generation (PMA + IFN-γ/LPS), APx-CcP^OE^ parasites again displayed increased infectivity compared to WT. Notably, in both strains, the presence of extracellular ascorbate significantly improved parasite survival, indicating that *T*. *cruzi* can utilize host ascorbate to fuel APx-CcP activity, counteracting peroxynitrite killing by macrophages (Fig. 5*C*).

## Discussion

Herein, we demonstrate that in a cell-free system, APx-CcP reacts fast with peroxynitrite (k ∼ 3–4 × 10^6^ M^-1^s^-1^ at pH 7.4, 25 °C; Fig. 1), at rates comparable to the fastest known hemeproteins involved in peroxynitrite detoxification (26, 31). The reaction most likely proceeds *via* a two-electron mechanism, forming a compound I-like intermediate (Fe^4+^=O, Trp233^•+^) and NO_2_^-^ (Fig. 2). Furthermore, APx-CcP was shown to decompose peroxynitrite catalytically using ascorbate (200 μM; Fig. 3). Still, to establish the relevance of APx-CcP in peroxynitrite detoxification *in cellula*, additional factors such as enzyme abundance, reducing substrate availability, and the presence of other peroxynitrite targets must be considered. Herein, we demonstrated that APx-CcP detoxifies macrophage-derived peroxynitrite (Fig. 4), an ability that promotes parasite survival through the neutralization of oxidative killing, facilitating phagosomal escape, and enhancing virulence in murine infections as previously shown (14).

Finally, the ability of *T*. *cruzi* to use extracellular ascorbate (at concentrations found in the mammalian host) as the reducing substrate for APx-CcP was assayed during infection to macrophages (Fig. 5). In naive macrophages and in the absence of added ascorbate, APx-CcP^OE^ parasites were more infective than WT, likely due to enhanced H_2_O_2_ detoxification. However, ascorbate addition did not further increase survival, possibly because of high levels of cytosolic ascorbate and the existence of other antioxidant systems (for diffusible H_2_O_2_) such as parasite peroxiredoxins (9, 41). In contrast, in immunostimulated macrophages (*i*.*e*. iNOS expression, ^•^NO and peroxynitrite production), ascorbate significantly improved the survival of both WT and APx-CcP^OE^ parasites, with the latter showing a stronger effect (Fig. 5). This finding aligns with the reported upregulation of APx-CcP during differentiation to the infective trypomastigote stage (42, 43), and consistent with the fact that WT parasites were also capable of peroxynitrite detoxification in the presence of ascorbate.

Altogether, our findings indicate that *T*. *cruzi* exploits the mammalian host cell ascorbate as reducing substrate for APx-CcP, enabling the fast detoxification of peroxynitrite and H_2_O_2_ in the phagosome. This mechanism likely contributes to parasite persistence through the mitigation of the macrophage-dependent oxidative damage in conjunction with other components of the enzyme antioxidant network in *T*. *cruzi*. In this regard, beyond the well-established role of thiol-dependent parasite peroxiredoxins in peroxide detoxification (k ∼ 10^6^–10^7^ M^-1^ s^-1^) and virulence in *T*. *cruzi* (5, 8, 9), APx-CcP constitutes a unique heme peroxidase with distinct subcellular localization and favorable reaction kinetics to cope against peroxynitrite. A previous report indicated that another hemeprotein from *Leishmania major* (referred to as a pseudoperoxidase (44)) was capable of decomposing peroxynitrite at moderate rates in the absence of a reducing substrate, probably through isomerization to nitrate and favoring the infection process *in cellula* and *in vivo*. Herein, we have established APx-CcP as a microbial heme peroxidase that catalytically and readily reduces peroxyntirite to nitrite at the expense of ascorbate and aids in the detoxification of macrophage-derived peroxynitrite to support parasite survival. Our results are also in agreement with animal experiments in which either *T*. *cruzi* APx-CcP^OE^ (14) or KO (45) cell lines promote or decrease infectivity *in vivo*, respectively. Thus, microbial heme-peroxidases such as APx-CcP must be viewed as a central part of the antioxidant armamentarium of pathogenic trypanosomatids.

## Experimental procedures

Unless otherwise specified, all assays were performed in 50 mM potassium phosphate buffer (pH 7.4) and at 25 °C. The concentrations of APx-CcP, H_2_O_2,_ and ONOO^-^ were spectrophotometrically determined at λ = 409 nm (101 mM^-1^ cm^-1^), λ = 240 nm (39.4 M^-1^ cm^-1^), and λ = 302 nm (1700 M^-1^ cm^-1^), respectively. Peroxynitrite was synthesized from sodium nitrite and H_2_O_2_ under acidic conditions in a quencher flow reactor as previously described (46) and stored at −80 °C. Briefly, equal volumes of freshly prepared H_2_O_2_ (0.6 M in HCl, 0.7 M) and sodium nitrite in water (0.6 M) are mixed at room temperature, and the flow (peroxynitrite) is rapidly quenched in NaOH (3 M). Excess H_2_O_2_ is removed by treatment with manganese dioxide (MnO_2_). The nitrite content was lower than 30% in all the peroxynitrite solutions.

### Expression and purification of recombinant APx-CcP in *E*. *coli*

The expression and purification of recombinant APx-CcP were performed as previously described (14). Briefly, *Escherichia coli* DE3 cells were transformed with the plasmid pTrcHis-APX (kindly provided by Dr Shane Wilkinson) and cultured in LB media, supplemented with ampicillin (100 μg mL^-1^) at 37 °C in a shaking incubator until reaching 0.6 AU at 600 nm. At this point, the temperature was lowered to 22 °C and protein expression was induced by the addition of isopropyl β-d-1-thiogalactopyranoside (IPTG, 0.5 mM) in the presence of γ-amino-levulinic acid (ALA, 0.5 mM). Supplementation of the culture medium with the heme precursor ALA significantly increases the yield of active recombinant protein (more than 60% heme incorporation). After overnight incubation, the protein expressed with a 6xHis-tag was purified by immobilized metal affinity chromatography (IMAC) using a column charged with Ni^2+^ (HiTrap 5 ml, Amersham Bioscience) equilibrated with binding buffer (50 mM phosphate buffer pH 7.4, 10 mM imidazole, and NaCl 500 mM). Elution was performed using a linear gradient of up to 500 mM imidazole at a flow rate of 3 ml min^-1^ in an ÄKTA pure protein purification system (GE Healthcare) with UV detection at 280 nm. Imidazole was immediately removed from the eluted fractions by gel filtration using a HiTrap desalting column (Amersham Biosciences) equilibrated with phosphate buffer 50 mM pH 7.4.

Protein purity was assessed by 12% SDS-PAGE and Coomassie Blue staining (Fig. S1). The percentage of heme incorporation in the purified samples was calculated from the A_409_/A_280_ ratio, considering molar absorption coefficients of 101 mM^-1^ cm^-1^ and 56.9 mM^-1^ cm^-1^ at 409 and 208 nm, respectively. The concentration determined from the 409 nm signal representing the holoprotein (65–70% heme incorporation) was used exclusively in all subsequent experiments.

### Kinetic studies of the APx-CcP reaction with peroxynitrite

#### Direct measurements

The reaction was monitored by the decomposition of peroxynitrite anion at 302 nm (ԑ_302_ = 1700 M^-1^ cm^-1^) in a SX20 stopped-flow spectrometer (Applied Photophysics, mixing time < 2 ms). The measurements were carried out with [Peroxynitrite]_0_ = 15 μM and [APx-CcP] variable concentrations from 0 to 10 μM. An initial rate approach was used to analyse the data as previously described (47). A linear function was fitted to the time courses during the first 10 ms of the reaction, and the initial rates were calculated by dividing the slope by the peroxynitrite molar extinction coefficient at 302 nm multiplied by a factor of 1.25 (47). This factor considers that at pH 7.4, 20% of peroxynitrite is in its neutral form (ONOOH), which does not absorb at the specified wavelength.

#### Competitive kinetic assay

The second-order rate constant of the APx-CcP reaction with peroxynitrite was alternatively determined by a competition assay using the boron-containing compound coumarin-7-boronic acid (CBA) (48). The hydroxylated fluorescent product, 7-hydroxycoumarin (COH), was measured by high-performance liquid chromatography (HPLC, Agilent Technologies 1200) coupled with fluorescence detection under the same conditions as previously described (35). Briefly, Samples (2 μl) were separated using an Ascentis Express Phenyl-Hexyl column (Supelco Analytical, 5 cm × 4.6 mm, 2.7 μm) equilibrated with 40% (v/v) acetonitrile (CH_3_CN) in 0.1% (v/v) trifluoroacetic acid aqueous solution. Isocratic elution was performed at a flow rate of 1 ml/min with fluorescence detection (λ_exc_ = 320 nm, _λem_ = 450 nm).

The second-order rate reaction was calculated as follows:

*k*_1_/*k*_2_ = ln([CBA]_0_/[CBA]_0_-[COH])/ln([APx-CcP]_0_/[APx-CcP]_0_-[APx-CcP]_ox_) where *k*_1_ is the reaction rate constant between CBA and peroxynitrite equal to 1.1 x 10^6^ M^-1^s^-1^ at pH 7.4 and 25 °C (49), and the initial concentrations of the reactants used in the assay were the following: [CBA]_0_ = 10 μM, [ONOO-]_0_ = 5 μM and [APx-CcP]_0_ = 7.5 to 20 μM.

To assess whether the enzyme can catalytically decompose the oxidant, a flux of peroxynitrite was generated with 3-morpholinosydnonimine (SIN-1, 0.5 mM) and Fluorescein-boronate (Fl-B) oxidation (10 μM) was followed over time in the presence of different APx-CcP concentrations (0.2, 2 and 4 μM) in the absence or presence of the reducing substrate ascorbate (200 μM). The time courses of Fl-B oxidation were monitored in a fluorescence plate reader at 37 °C (λ_exc_ = 492 nm and λ_em_ = 515 nm Varioskan, Thermo). To study a possible interference of ascorbate in the competition assay, Fl-B (10 μM) was mixed with a single dose (bolus addition) of peroxynitrite in the presence of variable concentrations of ascorbate (1, 2, 5, 10 and 20 mM) and Fl-B oxidation was monitored over time. As a positive control, a competition assay was performed in the presence of GSH (1, 2, 5, 10 and 20 mM), which reacts with peroxynitrite with k ∼1300 M^-1^s^-1^ (31).

#### UV-vis spectroscopic analysis

UV-visible spectra were recorded before and after mixing APx-CcP with peroxynitrite at a 1:1.5 ratio or H_2_O_2_ at a 1:1 ratio from 260 to 700 nm in a Shimadzu UV-visible spectrophotometer. Time-resolved absorbance spectra were recorded every 2 min for 25 min to assess the enzyme intermediate stability. The endogenous decay of the intermediate was monitored following the decrease in absorbance at 550 nm.

#### Immuno-spin trapping

Purified APx-CcP (10 μM) was exposed to peroxynitrite or H_2_O_2_ (10 or 30 μM) with vigorous vortex-mixing in the presence of the spin trap 5,5-dimethyl-1- pyrroline-N-oxide (DMPO, 100 mM). As previously shown, the alternative compound I generated upon oxidant addition (FeIV=O, Cys222^•^) can be trapped by DMPO generating a stable protein-DMPO nitrone adduct that can be detected by Western blot with specific anti-DMPO-nitrone antibodies (33).

Alternatively, the enzyme was treated with N-ethyl-maleimide (NEM, 50 mM) before the assay to alkylate the sulfhydryl at Cys222. This alkylation inhibits the “alternative” compound I generation (see Fig. 2*B*). The reaction was performed at room temperature. After treatment, proteins were subjected to 12% SDS-PAGE under reducing conditions and then analyzed by western blotting as described above. Protein-DMPO adducts were detected using a chicken polyclonal anti-DMPO-nitrone primary antibody (kindly provided by Ronald Mason, National Institutes of Environmental Health Sciences) (33) and rabbit polyclonal anti-*T*. *cruzi* APx-CcP antibody (1:1000) previously validated in (14).

#### Western blot

After treatment, proteins were subjected to 12% SDS-PAGE under reducing conditions, transferred onto a nitrocellulose membrane, and stained with Ponceau S solution. The membranes were treated with a blocking solution (PBS 50 mM, pH 7.4, containing dry milk 5% w/v) for 1 h at RT, followed by overnight incubation at 4 °C with mouse monoclonal anti-3-nitrotyrosine (1:2000), rabbit polyclonal anti-*T*. *cruzi* APx-CcP (1:1000, (14)), mouse anti-cMyc (1:1000, ab32 Santa Cruz) or chicken polyclonal anti-DMPO-nitrone antibodies in PBS containing Tween 20 (0.1% v/v). The membranes were washed and probed for 1 h at RT with conjugated secondary antibodies (IR Dye-800- and IR Dye- 680-conjugated, LI-COR Biosciences) diluted 1:15,000 in PBS containing Tween 20 (0.1% v/v). After washing, the membranes were visualized on an infrared fluorescence detection system (Odyssey, LI-COR Biosciences).

#### Nitrite determination

APx-CcP (0–100 μM) was exposed to peroxynitrite at a 0.5:1 protein: oxidant ratio in buffer phosphate (50 mM, pH 7.4). A standard curve was generated with NaNO_2_ (0–100 μM) in the presence of APx-CcP (50 μM). After oxidant addition, samples were precipitated with methanesulfonic acid (4 M) for 30 min at 4 °C, following centrifugation at 13000 x g at 4 °C to remove APx-CcP. The supernatants (100 μl) were then mixed with an equal volume of the Griess reagent, and the absorbance was read at 540 nm.

#### Peroxynitrite-mediated enzyme inactivation and nitration

Purified APx-CcP (15 μM) was treated with peroxynitrite (0–2000 μM) added as a single bolus under vigorous vortexing, and residual enzyme activity and levels of tyrosine nitration were evaluated. Enzyme activity assays using either ascorbate or horse cytochrome c as a reducing substrate were carried out as previously described (14). Briefly, ascorbate-dependent peroxidase activity was monitored at 290 nm (extinction coefficient of 2800 M^-1^ cm^-1^) using APx-CcP (0.2 μM) in the presence of H_2_O_2_ (40 μM) and ascorbate (200 μM). The Cc-dependent peroxidase activity was measured by monitoring ferrocytochrome c (Cc^2+^) oxidation at 550 nm using APx-CcP (0.2 μM) in the presence of H_2_O_2_ (40 μM) and Cc^2+^ (50 μM). Additionally, the levels of tyrosine nitration were analyzed by western blotting using a primary antibody against protein 3-nitrotyrosine, as described above.

#### Cell culture conditions

*T*. *cruzi* epimastigotes (CL-Brener, wild-type; WT) were cultured at 28 °C in brain heart infusion (BHI) containing heat inactivated bovine fetal serum (10% v/v) (50). *T*. *cruzi* epimastigotes overexpressing *Tc*APx-CcP were obtained as previously described (9, 15). Transformed *Tc*APx-CcP (CL-Brener-pTEX-APX-9E10) overexpressing epimastigotes were cultured in BHI medium supplemented with G418 (250 μg/ml; Sigma). Parasites were differentiated to the infective metacyclic stage and metacyclic forms purified by overnight incubation with fresh human serum as previously described (9). Metacyclic trypomastigotes were used to infect confluent Vero cells (ATCC) at 37 °C in a 5% CO_2_ atmosphere. Culture-derived trypomastigotes were collected by centrifugation of the supernatant of infected Vero cells at 4000 x g at room temperature for 10 min and incubated for 1 h at 37 °C, to allow viable parasites to move from the pellet into the supernatant. The murine macrophage cell line J774A.1 was cultured in DMEM (Sigma) supplemented with L-glutamine (2 mM), penicillin (100 units/ml), streptomycin (100 mg/ml), and heat-inactivated fetal bovine serum (10% v/v) at 37 °C in a 5% CO_2_ atmosphere. For the induction of iNOS expression, macrophages were immuno-stimulated (IFN-γ, 800 UmL^-1^ and LPS, 16 μg/ml) for 5 h and ^•^NO production was assayed by detecting nitrite (NO_2_^-^) accumulation 24 h later in the cell culture supernatant by the Griess method (5). The macrophages used in all the experiments were passaged fewer than 15 times.

### Macrophage-derived oxidant detection

Macrophages seeded in 24-well plates with or without immuno-stimulation were incubated in Dulbecco’s PBS (dPBS) in the presence or absence of ascorbate (200 μM). Fl-B (50 μM) was then added and O_2_^•-^ production by NOX-2 was stimulated by the addition of phorbol 12-myristate 13-acetate (PMA, 4 μg/ml). Fl-B oxidation by H_2_O_2_ and/or peroxynitrite was monitored using a microplate reader at 37 °C (λ_ex_ = 492 nm and λ_em_ = 515 nm; Varioskan, Thermo). Intraphagosomal oxidant detection was performed with Fl-B pre-loaded culture-derived trypomastigotes as previously (35). Briefly, trypomastigotes (5 x 10^5^ cells mL^−1^) were pre-loaded with Fl-B (100 μM) for 30 min at 37 °C in dPBS. Following incubation, trypomastigotes were centrifuged at 800 g for 10 min at 25 °C and washed twice in dPBS to eliminate non-incorporated probe. Pre-loaded parasites were incubated with control or immuno-stimulated macrophages (parasite: cell ratio of 5:1) in the different experimental conditions (presence or absence of: L-NAME, 10 mM; ascorbate, 200 μM; NOC-18, 0.5 mM) and after 1 h of incubation, Fl-B oxidation was monitored using a microplate reader at 37 °C (Varioskan, Thermo) as above or macrophages were trypsinized and analyzed by flow cytometry (FACS Calibur; Becton Dickinson). At least 30,000 cell events were recorded in each experimental treatment and analyzed using WinMID 2.9.

### Macrophage *T*. *cruzi* infection

Macrophages were seeded in Lab-Tek chamber slides and immunostimulated or left untreated for 5 h. Macrophages where then infected with culture-derived trypomastigotes from WT (CL-Brener) and *Tc*APx-CcP-overexpressing parasites (parasite:cell ratio of 5:1) for 2 h in the presence or absence of ascorbate (200 μM, freshly prepared). Non-engulfed parasites were removed by washing twice in dPBS (Dulbecco’s PBS, pH 7.4; Sigma), and cells were further incubated for 24 h in DMEM at 37 °C. Infected cells were fixed in a 4% (v/v) fresh paraformaldehyde solution in PBS for 30 min at room temperature, washed with PBS, and permeabilized for 5 min with 0.1% (v/v) Triton X-100 in PBS. The number of parasites per 100 macrophages was determined by DAPI staining (5 μg/ml). Preparations were analyzed using a microscope (Nikon Eclipse TE-200) at x1000 magnification, and digital photographs of infected cells were recorded. At least 2500 cells from four independent experiments were counted in duplicates. Results represent the mean ± SD and are expressed relative to WT infection in control conditions (naïve macrophages) considered as 1.

### Statistical analysis

All the data are presented as means ± SD of at least 3 to 4 independent experiments. Means were compared by one-way ANOVA followed by Tukey's multiple comparison test. A *p-*value of < 0.05 was considered statistically significant.

## Data availability

All data are contained within the manuscript.

## Supporting information

This article contains supporting information.

## Conflict of interest

The authors declare that they have no conflicts of interest with the contents of this article.
