# Supplementary material for: Peroxynitrite detoxification by Trypanosoma cruzi heme peroxidase supports parasite survival in macrophages
Source: J Biol Chem. 2025 Jul 28;301(9):110533. doi: 10.1016/j.jbc.2025.110533 (PMC12398798; doi:10.1016/j.jbc.2025.110533)
Supplement: Supplementary Material [file mmc1.docx]

**Peroxynitrite detoxification by *Trypanosoma cruzi* heme peroxidase supports parasite survival in macrophages**

Vera Skafar^1,2^, Matilde Abboud^1,2^, Samuel Freeman^3^, Alejandra Martínez^1,2^, Emma L. Raven^3*^, Rafael Radi^1,2*^ and Lucía Piacenza^1,2*^

 1 2 3 4 5 6 7 8 9 10 APxCcP MW (kDa)

**Figure S1. Purification of recombinant APx-CcP**. SDS-PAGE (12%) analysis of the fractions eluted from the Ni^2+^-affinity chromatography (see materials and methods). Only pure fractions (7-10) were used for the experiments. Pooled and concentrated recombinant APx-CcP was transferred to nitrocellulose membranes and stained with Ponceau S, the molecular weight of the recombinant enzyme is 35.6 kDa as expected. MW: molecular weight (kDa).

| **APx-CcP**  **(μM)** | **ONOO^-^**  **(μM)** | **NO_2_^-^**  **(μM) ± SD** | **Yield (%)** |
| --- | --- | --- | --- |
| 55 | 100 | 47.4 ± 6 | 86 |
| 55 | 200 | 58 ± 9 | 103 |
| 70 | 140 | 60.3 ± 5 | 86 |

**Table S2. Nitrite determination by the Griess reaction following peroxynitrite addition to APx-CcP.** The yield was calculated considering that all the initial APx-CcP reacts with peroxynitrite by a two-electron reduction mechanism. Each NO_2_^-^ determination is the mean value ± standard deviation (SD) of three independent experiments.

**Figure S2. Resistance of APx-CcP to peroxynitrite inactivation.** **a**. Cytochrome c peroxidase (CcP) or **b**. Ascorbate peroxidate (APx) activity of APx-CcP (15 µM) versus peroxynitrite concentration (0-2 mM) in buffer phosphate (50 mM, pH 7.4). The enzymatic activity was measured using cytochrome c (left) or ascorbate (right) as reducing substrates. Oxidation of ferrocytochrome c (Cyt c^+2^) was followed at 550 nm whereas ascorbate oxidation was followed at 290 nm. Activity is expressed as 100% in control condition (absence of peroxynitrite). Inset: magnification of the activity up to 100 µM peroxynitrite **c**. Nitro-tyrosine (NO_2_-Tyr) detection by western blot analysis using polyclonal anti-NO_2_-Tyrosine antibodies (1:2000) in APx-CcP exposed to different concentrations of peroxynitrite (0-1.5 mM).
